# Laparoscopic Splenectomy for Inflammatory Pseudotumor‐Like Follicular Dendritic Cell Sarcoma: A RareEpstein–Barr Virus‐Associated Splenic Tumor

**DOI:** 10.1002/kjm2.70082

**Published:** 2025-08-04

**Authors:** Hsin‐Yi Lin, Po‐Hsuan Wu, Hui‐Gin Chiu, Jia‐Yu Chen

**Affiliations:** ^1^ Department of Education China Medical University Hospital Taichung Taiwan; ^2^ Division of General and Digestive Surgery Kaohsiung Medical University Hospital, Kaohsiung Medical University Kaohsiung Taiwan; ^3^ College of Medicine Kaohsiung Medical University Kaohsiung Taiwan; ^4^ Division of General and Digestive Surgery Kaohsiung Medical University Gangshan Hospital Kaohsiung Taiwan

Dear editor,

Inflammatory pseudotumor‐like follicular dendritic cell sarcoma (IPT‐like FDCS) is a rare, indolent neoplasm regarded as a histological variant of FDCS, typically originating from hematopoietic and lymphoid tissues [1, 2]. FDCS typically presents with nonspecific clinical manifestations and is morphologically divided into conventional and IPT‐like FDCS [2, 3]. Unlike conventional FDCS, which primarily arises in the lymph nodes, IPT‐like FDCS nearly exclusively occurs in extranodal organs—most commonly the liver and the spleen [1, 4]—and is strongly associated with Epstein–Barr virus (EBV) infection [2, 4]. Given its nonspecific clinical presentation and lack of distinctive radiologic features, preoperative diagnosis is often difficult. Herein, we report a case of a 64‐year‐old woman with splenic IPT‐like FDCS successfully treated with laparoscopic splenectomy. No recurrence was observed during a 2‐year follow‐up.

A 64‐year‐old woman without systemic disease was referred to our hospital after the incidental detection of a splenic mass during routine abdominal sonography. Despite generalized weakness, her symptoms were not specific. Laboratory results and tumor markers (carcinoembryonic antigen, squamous cell carcinoma antigen, and cancer antigen 125) were within normal limits. Abdominal computed tomography showed a well‐defined hypodense splenic mass with central low attenuation suggesting necrosis (Figure 1A). Laparoscopic splenectomy was performed. Grossly, the tumor was a solitary, well‐circumscribed mass (6.0 × 5.5 × 5.0 cm) that arose in the upper pole of the spleen, was firm, and tan‐white with focal hemorrhage and necrosis (Figure 1B). Histological examination revealed spindle‐shaped cells with bland nuclei and mild atypia in fascicular and storiform patterns, surrounded by dense lymphoplasmacytic infiltrates. The stroma contained collagen bundles and focal fibrin, with no marked mitotic activity or capsular invasion (Figure 1C,D). Cytological smears revealed loosely cohesive spindle‐to‐oval cells with vesicular chromatin. Immunohistochemistry demonstrated diffuse CD21 (Figure 1E) and CD23 positivity. EBV‐encoded RNA (EBER) in situ hybridization confirmed association with EBV (Figure 1F). The final diagnosis was IPT‐like FDCS. The patient recovered well postoperatively and had no recurrence at the 2‐year follow‐up.

**FIGURE 1:**
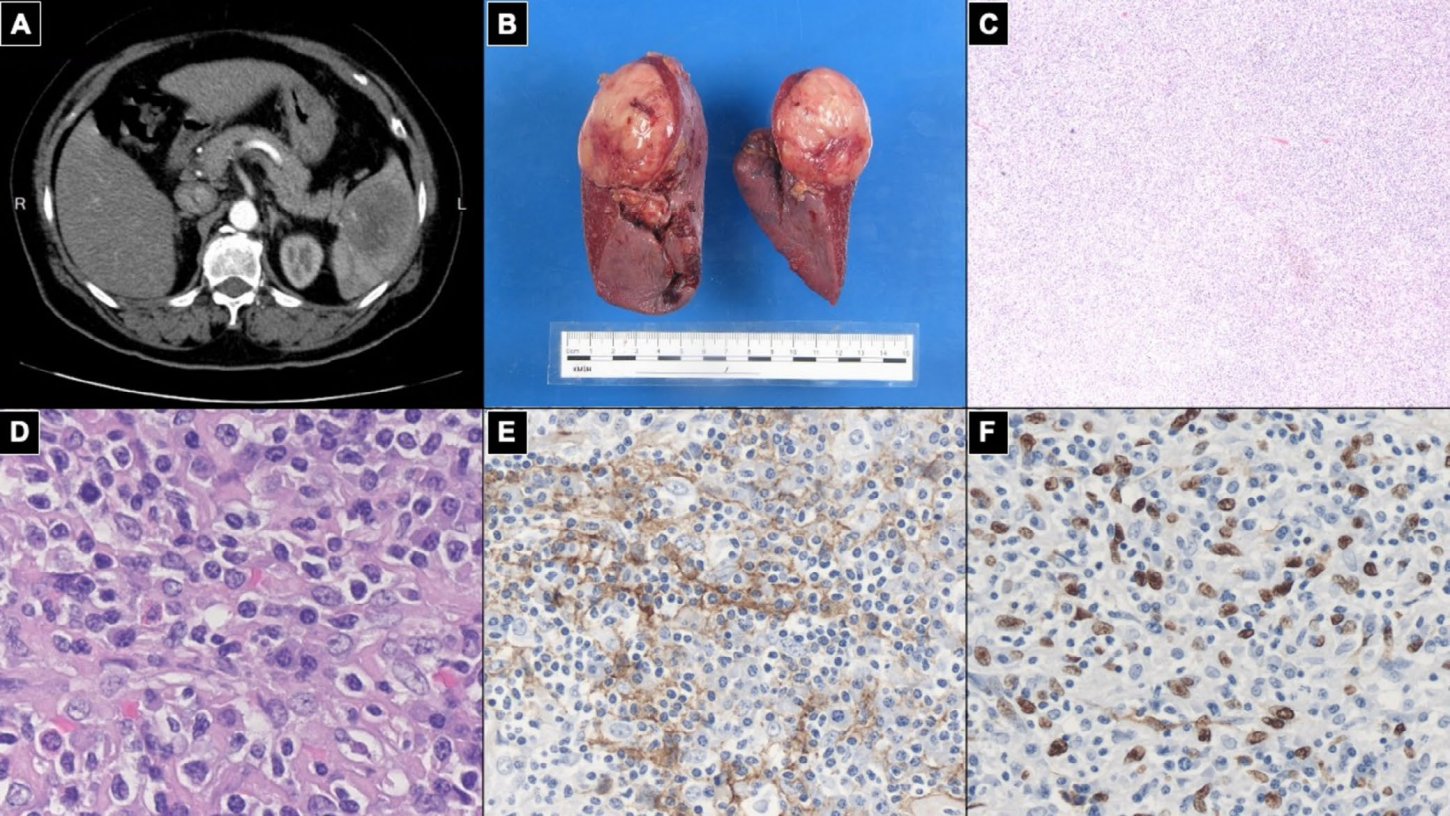
(A) Axial CT scan showing a well‐demarcated, hypodense lesion in the spleen with central necrosis and no signs of adjacent organ invasion. (B) Gross appearance of the resected spleen, revealing a 6.0 × 5.5 × 5.0 cm well‐circumscribed, tan‐white mass with focal hemorrhagic and necrotic areas at the upper pole. (C) Low‐power view (H&E, 40×) showing the spindle cell lesion embedded in a prominent inflammatory background with diffuse lymphoplasmacytic infiltration. (D) High‐power view (H&E, 200×) demonstrating bland spindle cells with mild atypia arranged in fascicular and storiform patterns, admixed with plasma cells and lymphocytes. (E) Immunohistochemistry for CD21 (200×) showing strong membranous and dendritic staining in tumor cells, consistent with follicular dendritic cell origin. (F) EBER in situ hybridization (200×) highlighting nuclear positivity in spindle tumor cells, confirming Epstein–Barr virus association.

IPT‐like FDCS is a rare, slow‐growing tumor that mainly affects middle‐aged to older people, with women making up approximately 60%–62% of cases [1, 2]. Fewer than 100 cases involving the liver and spleen have been reported globally, with > 80% occurring in East Asia, probably due to regional EBV rates and social transmission patterns [3]. Most tumors are found accidentally during routine imaging or present with nonspecific symptoms such as abdominal pain, discomfort, or thrombocytopenia [4, 5]. The tumors can range widely in size—from 2 cm to as large as 20 cm—although most are < 6 cm, with occasional reports of tumors measuring > 10 cm [5].

EBV is strongly associated with IPT‐like FDCS and is crucial in its development. The virus infects follicular dendritic cells via the CD21 receptor, leading to increased expression of CD21 and CD35. The consistent detection of IgG4^+^ plasma cells in Asian splenic cases indicates a potential EBV–IgG4 immune axis that may affect tumor structure and regional occurrence. Despite the rarity of EBV‐negative cases, EBV is likely the primary oncogenic trigger in most cases.

Diagnosis is challenging owing to its nonspecific clinical features that overlap with inflammatory pseudotumors, lymphomas, and conventional FDCS. Immunohistochemistry and EBER in situ hybridization are critical. CD21 is the most consistent marker, with variable expression of CD23 and CD35 [2]. The present case was positive for CD21/CD23 and had strong EBER staining, confirming EBV association.

Radiologically, IPT‐like FDCS typically presents as a well‐defined, hypodense lesion with possible central necrosis or calcification but lacks specific imaging features [5]. Histologically, the tumor consists of spindle to ovoid cells in storiform or fascicular patterns within a dense inflammatory stroma [5].

Surgical resection remains the main treatment. Laparoscopic splenectomy, as performed in the presented case, is effective and associated with favorable recovery [1]. Systemic therapy is generally reserved for unresectable or metastatic disease [2]. Few cases have explored systemic therapies, including chemotherapy and immunotherapy; however, data are limited, and long‐term efficacy remains uncertain [2]. Compared with conventional FDCS, IPT‐like FDCS has a better prognosis, with recurrence rates of 9.6%–16.7%, a metastasis rate of approximately 15.9%, and a mortality rate as low as 2.9% [1, 2, 3]. The presented case reinforces the favorable outcome with complete resection.

## Ethics Statement

All procedures performed in studies involving human participants were in accordance with the ethical standards of the institutional and/or national research committee and with the 1964 Helsinki declaration and its later amendments or comparable ethical standards.

## Conflicts of Interest

The authors declare no conflicts of interest.

## Data Availability

Data sharing not applicable to this article as no datasets were generated or analysed during the current study.
